# Nigrostriatal neuronal death following chronic dichlorvos exposure: crosstalk between mitochondrial impairments, α synuclein aggregation, oxidative damage and behavioral changes

**DOI:** 10.1186/1756-6606-3-35

**Published:** 2010-11-13

**Authors:** Binukumar BK, Amanjit Bal, Ramesh JL Kandimalla, Kiran Dip Gill

**Affiliations:** 1 Department of Biochemistry, Postgraduate Institute of Medical Education and Research, Chandigarh, 160012, India; 2 Department of Histopathology, Postgraduate Institute of Medical Education and Research, Chandigarh, India

## Abstract

**Background:**

In recent years, several lines of evidence have shown an increase in Parkinson's disease prevalence in rural environments where pesticides are heavily used. Although, the underlying mechanism for neuronal degeneration in sporadic PD remains unknown, mitochondrial dysfunction, oxidative stress and proteasomal dysfunction are proposed as contributing factors. In this study rats were chronically and continuously exposed to the pesticide, dichlorvos to identify the molecular mechanism of nigrostaital neuronal degeneration.

**Result:**

Chronic dichlorvos exposure (2.50 mg/kg b.wt.s.c/daily for 12 weeks) caused nigrostriatal dopaminergic degeneration. The degenerative changes were accompanied by a loss of 60-80% of the nigral dopamine neurons and 60-70% reduction in striatal dopamine and tyrosine hydroxylase levels. Dichlorvos exposed animals also showed α -synuclein and ubiquitin positive inclusions along with swollen, dystrophic neurites and mitochondrial abnormalities like decreased complex I&IV activities, increased mitochondrial size, axonal degeneration and presence of electron dense perinuclear cytoplasmic inclusions in the substantia nigra of rats. These animals also showed evidence of oxidative stress, including increased mitochondrial ROS levels, decreased MnSOD activity and increased lipid peroxidation. Measurable impairments in neurobehavioral indices were also observed. Notable exacerbations in motor impairments, open field and catalepsy were also evident in dichlorvos exposed animals.

**Conclusion:**

All these findings taken together indicate that chronic dichlorvos exposure may cause nigrostaital neurodegenaration and significant behavioral impairments.

## Background

Parkinson's disease (PD) is a common neurodegenerative disease characterized by disabling motor abnormalities, which include tremors, muscle stiffness, paucity of voluntary movements, and postural instability [1]. Its main neuropathological feature is the loss of the nigrostriatal dopamine containing neurons, whose cell bodies are in the substantia nigra pars compacta (SNpc) and nerve terminals in the striatum. Epidemiological studies indicate that pesticides are one of the leading candidates of environmental toxins that may contribute to the pathogenesis of PD [2,3]. Reports of parkinsonism following pesticide exposure [4-6] make pesticide-induced parkinsonism biologically plausible. Hertzman et al [4] found a significant association between PD and an occupation of handling pesticides in British Columbia. PD is most prevalent in industrialized countries [7]. For example in China, the ubiquity of PD is much lower as compared to the more industrialized USA. However, even in China, PD appears to be associated with exposure to industrial chemicals [7]. For example in China, the ubiquity of PD is much lower as compared to the more industrialized USA [6]. However, even in China, PD appears to be associated with exposure to industrial effluents [7], as observed by Ho et al [8] who found that subjects previously exposed to herbicides/pesticides had a 3.6-fold increased risk of developing PD in Hong Kong.

Although, the underlying mechanism for neuronal degeneration in sporadic PD remains unknown, mitochondrial dysfunction, oxidative stress and proteasomal dysfunction are proposed as contributing factors. Rotenone, a common pesticide and an inhibitor of mitochondrial complex I, has been shown to induce Parkinsonian features in rats [3]. Other pesticides, such as paraquat and dieldrin, have also been reported to cause degeneration of dopaminergic neurons [9]. The mechanisms however, remain largely unknown. Although, the majority of PD patients have sporadic onset and do not have a familial history, genetic findings have provided important insights into pathogenic mechanisms of PD [10]. The causes of this disease still remain to be elucidated. Understanding the cause of PD is critical as that knowledge could lead to directed research that will develop potent therapies. The relative contributions of genetic versus environmental factors regarding the cause of PD have been hotly debated. In this contest, we thought of exposing rats chronically and continuously to the pesticide dichlorvos (DDVP) which is one of the most commonly used organophosphate (OP) pesticide in India and studying its mechanism of action. It has been commercially manufactured and used throughout the world. It is also an inhibitor of complex I, one of the five enzyme complexes of the inner mitochondrial membrane [11]. Because of its extremely hydrophobic nature, it crosses biological membranes easily, and it does not depend on the dopamine transporter for access to the cytoplasm. Recently, different environmental toxins have been investigated that take into account epidemiological and environmental risk factors for PD. In this study, one basic question asked was whether nigrostriatal neuronal degeneration and Lewy body formation are produced after chronic low level dichlorvos exposure? And if yes, what are its consequences? In addition, the relevance of mitochondrial dysfunctions to potential involvement of chronic low level dichlorvos exposure leading to behavioral changes was also kept in mind. At the end of the study we found that, chronic dichlorvos exposure causes marked nigrostriatal neuronal degeneration along with Lewy body formation and mitochondrial impairments leading to behavioral changes in rats.

## Result

Male albino rats (Wistar strain) were administrated dichlorvos (2.5 mg/kg, sc. dissolved in corn oil over a period of 12 weeks systemically on a daily basis). In this study, pulsatile administration was chosen because it is more close to exposure in normal life, such as via inhalation, dermal contact, and the oral ingestion of pesticide residues, via foods such as vegetables, fruits and fish. High doses of dichlorvos for short periods of time produced systemic (cardiovascular and liver) toxicity and non-specific brain lesions, as reported earlier from our lab and others [11-14]. Downward titration of dichlorvos dosing resulted in less systemic illness and dopaminergic degeneration. We wanted to choose a low dose of dichlorvos, which is similar to human exposure especially in rural areas where pesticides are in more use, and also factories where pesticides are manufactured. As far as rural area and pesticide factories are concerned persons working there are exposed to pesticides slowly at lower doses and for long durations. Three month exposure of dichlorvos to rats is equivalent to approximately seven and half years of human exposure. At this very dose regime it was observed that acetylcholine esterase activity was not inhibited and no signs of systemic toxicity were observed in these animals.

### Studies on the effect of dichlorvos induced mitochondrial impairments on dopaminergic system in substantia nigra and corpus striatum of rat brain

Mitochondrial dysfunction has also been linked to PD. Specifically, there are systemic reductions in the activity of complex I and 1V of the mitochondrial ETC in PD brain, muscle and additional evidence for mitochondrial impairment in PD comes from the finding that MPP, the active metabolite of the parkinsonism toxin MPTP, acts as a complex I inhibitor. Therefore, it was pertinent to investigate the different aspects of mitochondrial energy metabolism *in vivo *in the wake of chronic dichlorvos exposure in different rat brain regions (SN and CS).

### Effect of chronic dichlorvos exposure on NADH dehydrogenase activity in substantia nigra and corpus striatum of rat brain

**Figure 1:**
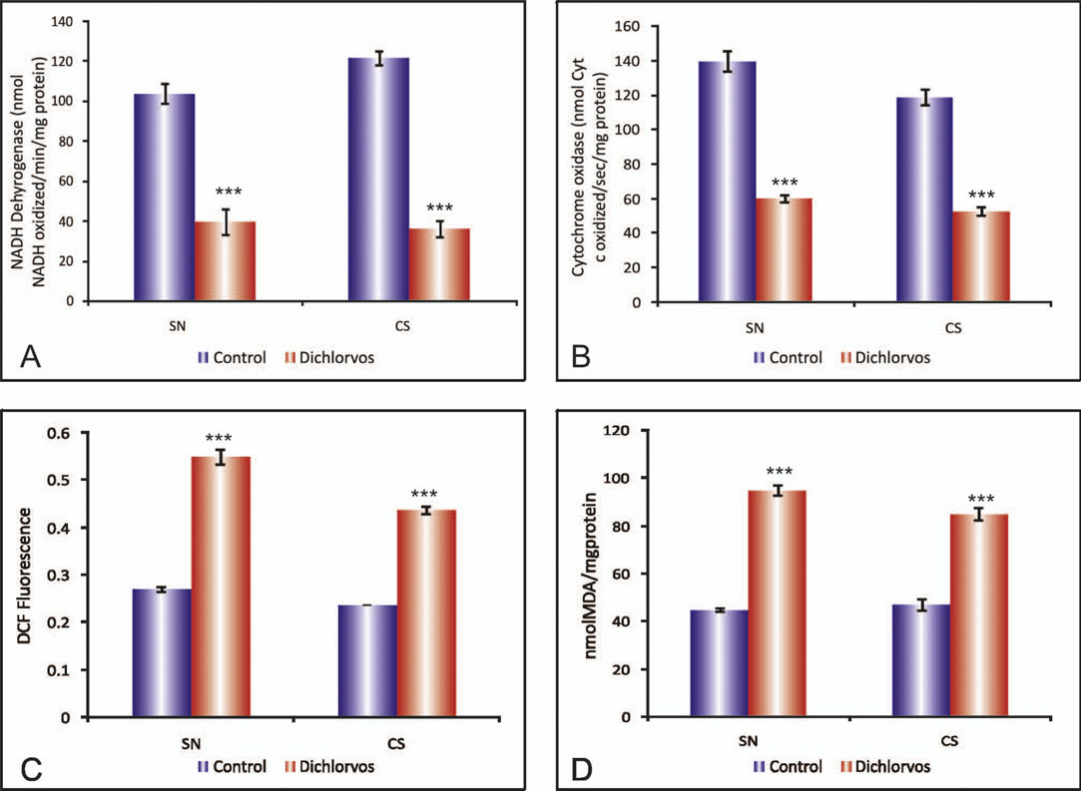
**Effect of chronic dichlorvos exposure on mitochondrial electron transport chain**.**(A**) NADH dehydrogenase activity. (**B) **Cytochrome oxidase activity. (**C**) ROS levels. (**D**) Lipid peroxidation. Dichlorvos treated rats received 2.5 mg/kg b.wt of dichlorvos, sc., for 12 weeks and control animals received equal volume of corn oil. The values are mean ± S.D. of 6 animals in each group. *** p < 0.001 significantly different from control group.SN: substantia nigra; CS: corpus striatum.

NADH dehydrogenase or complex I is a membrane bound enzyme, which catalyzes the transfer of two electrons from NADH to ubiquinone in a reaction that is associated with proton translocation across the membrane. Any alteration in the activity of NADH dehydrogenase may disturb the in flow of electrons through various electron carriers in the electron transport chain. Therefore, the activity of NADH dehydrogenase was assessed in rat brain mitochondria following chronic dichlorvos exposure. It is clear from Figure 1A, that there was a significant decrease in the activity of NADH dehydrogenase in both the regions of rat brain following chronic dichlorvos administration. Brain mitochondrial NADH dehydrogenase activity was decreased by 62% in SN followed by CS (70.2%) in dichlorvos treated animals as compared to controls (Figure 1A).

### Effect of chronic dichlorvos exposure on cytochrome oxidase activity in substantia nigra and corpus striatum of rat brain

Cytochrome oxidase (Complex IV) is the terminal enzyme of the mitochondrial electron transport chain and is responsible for the reduction of molecular oxygen, which is linked to ATP production. In view of this, the activity of cytochrome oxidase was assayed in rat brain mitochondria. Cytochrome oxidase activity was decreased by 56.7%, in SN of dichlorvos treated animals as compared to controls (Figure 1B). Further, the mitochondria isolated from CS of rat brain showed 55.4% decrease in the activity of complex IV following 12 weeks of dichlorvos exposure (Figure 1B). This decrease was found to be highly significant in both the regions (p < 0.001) of treated rat brain mitochondria. The decrease in the activity of cytochrome oxidase could be responsible for the ability of dichlorvos to interfere with oxygen metabolism and the possible generation of the state of cellular hypoxia as well as in the production of oxygen free radicals.

It appears that dichlorvos consistent with its ability to cross biological membranes because of its lipophilicity had a significant detrimental effect on complex 1 and IV which are embedded in the mitochondrial membrane.

### Effect of dichlorvos on Mitochondrial ROS levels in substantia nigra and corpus striatum of rat brain

Mitochondria are the major cellular source of intracellular ROS. In our study it was observed that dichlorvos inhibited Complex I and IV activities, and therefore it may also result in enhanced ROS production. Therefore ROS levels were measured using the dye 2,7-dichlorofluorescein diacetate. As compared to controls, the mitochondria isolated from the brains (SN and CS regions) of dichlorvos exposed animals showed significant increase (p < 0.001) in signal fluorescence suggesting higher ROS accumulation in mitochondria of these regions (Figure 1C). Our results suggest that increased rate of H_2_DCF oxidation be interpreted as an indication of general oxidative stress due to a variety of reasons, including depletion of antioxidants, rather than as a specific proof of augmented ROS formation.

These results further substantiate the findings that chronic dichlorvos exposure leads to mitochondrial dysfunction by acting at Complex 1 and IV of electron transport chain. These impairments of mitochondrial functions may be involved in the generation of ROS and hence a state of oxidative stress.

### Effect of dichlorvos on Lipid peroxidation in substantia nigra and corpus striatum of rat brain

Oxidative stress has been considered as an important factor in the pathogenesis of PD. To study the potential involvement of oxidative stress in dichlorvos neurotoxicity, we measured lipid peroxidation by measuring MDA accumulation. It was significantly increased in the SN and CS after chronic dichlorvos treatment (Figure 1D). In SN lipid peroxidation levels were 50.29% higher as compared to control SN region. In the case of CS region of dichlorvos exposed animal, 38% increased lipid peroxidation was observed when compared to control CS region.

### Effect of chronic dichlorvos exposure on Mn SOD activity in substantia nigra and corpus striatum of rat brain

Mitochondria are the main intracellular source of free radical generation. Superoxide dismutase (SOD) is present in all aerobic cells. MnSOD an isoform of SOD, is exclusively mitochondrial in origin and functions to facilitate the dismutation of superoxide radicals to H_2_O_2_. Results of SOD activity are shown in additional file 1. The mean MnSOD activity was 25.78 ± 5.27 U/mg protein and 30.08 + 5.2 U/mg protein in SN and CS respectively in the control animals. Where as MnSOD activity in the animals treated with dichlorvos (SN 10.5 + 3.5 and CS 15.94 + 3.67), showed statistically significant decrease (after 12 weeks). A decreased MnSOD activity reveals a state of oxidative stress in the mitochondria of dichlorvos exposed rats.

### Effect of chronic dichlorvos exposure on ATP Levels in substantia nigra and corpus striatum of rat brain

**Figure 2:**
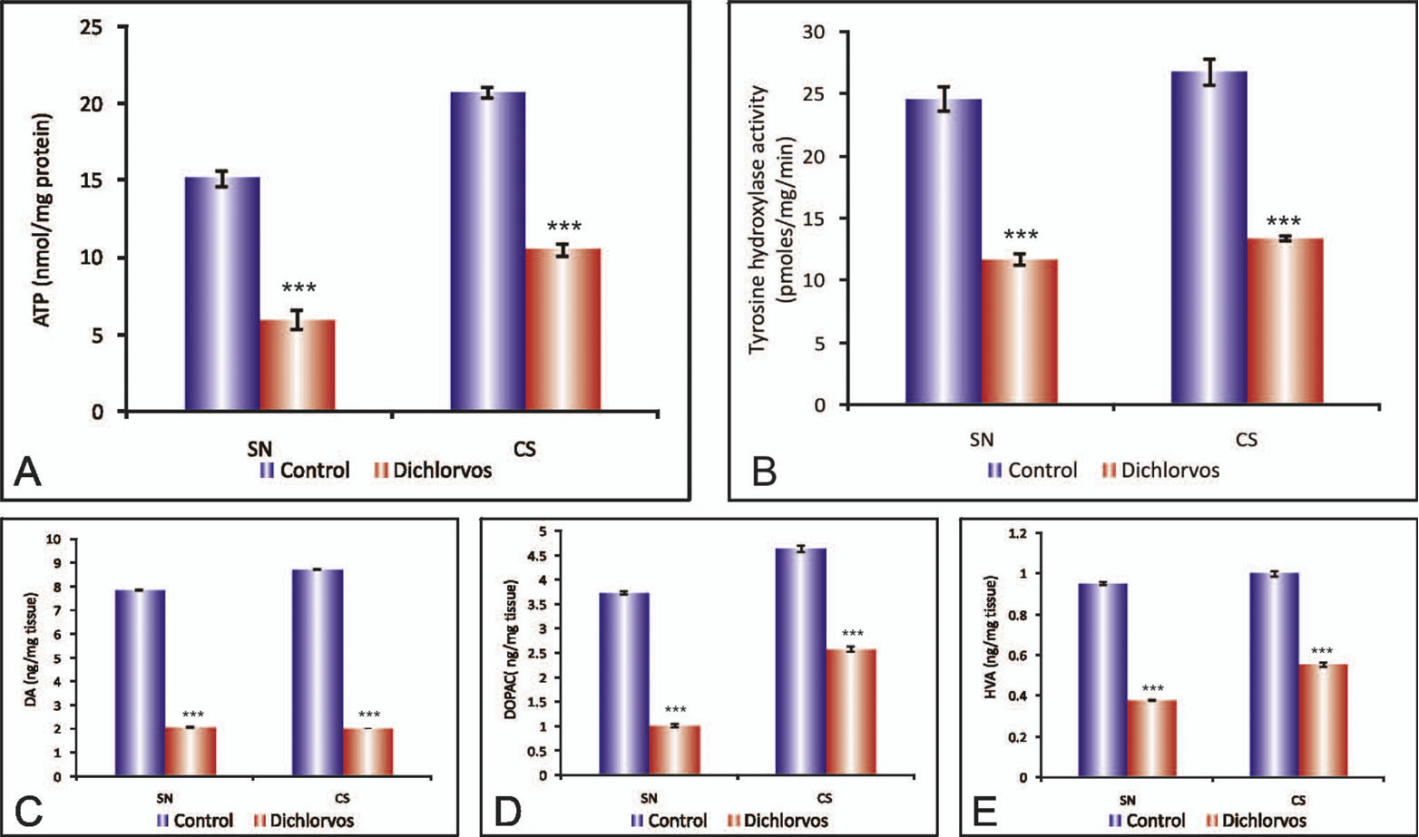
**Effect of chronic dichlorvos exposure on ATP levels, Tyrosine hydroxylase activity, Dopamine and its metabolites in SN and CS of rat brain**. **(A) **ATP levels. (B). Tyrosine hydroxylase activity (C). Dopamine levels. (D) DOPAC levels. (E) HVA levels. The values are mean ± S.D. of 6 animals in each group. *** p < 0.001 significantly different from control group. SN: substantia nigra; CS: corpus striatum.

As the primary energy source of most cells, ATP generation is essential to cell survival; impairment of mitochondrial respiration implies the reduced availability of ATP for maintaining essential cellular processes. As we had observed inhibition of respiratory complexes as well as MnSOD and increased generation of ROS and lipid peroxidation, it was imperative to estimate the ATP levels so as to assess the energy status of the cell. The ATP levels in brain mitochondria were estimated following chronic dichlorvos exposure. As evident from Figure 2A, the ATP levels declined significantly in different regions of dichlorvos treated rats. The level of ATP decreased by 60.8% in SN of treated group, portraying altered mitochondrial metabolism in this region. The decrease in ATP levels in the other region viz CS was 49%. This observation suggested that cells exposed to dichlorvos suffered from energy deficit and a state of chronic persistent energy crisis might have been generated.

### Studies on the ultra structural changes of mitochondria in SN and CS neurons after chronic dichlorvos exposure

#### Mitochondrial abnormalities in the SN after chronic dichlorvos exposure

**Figure 3:**
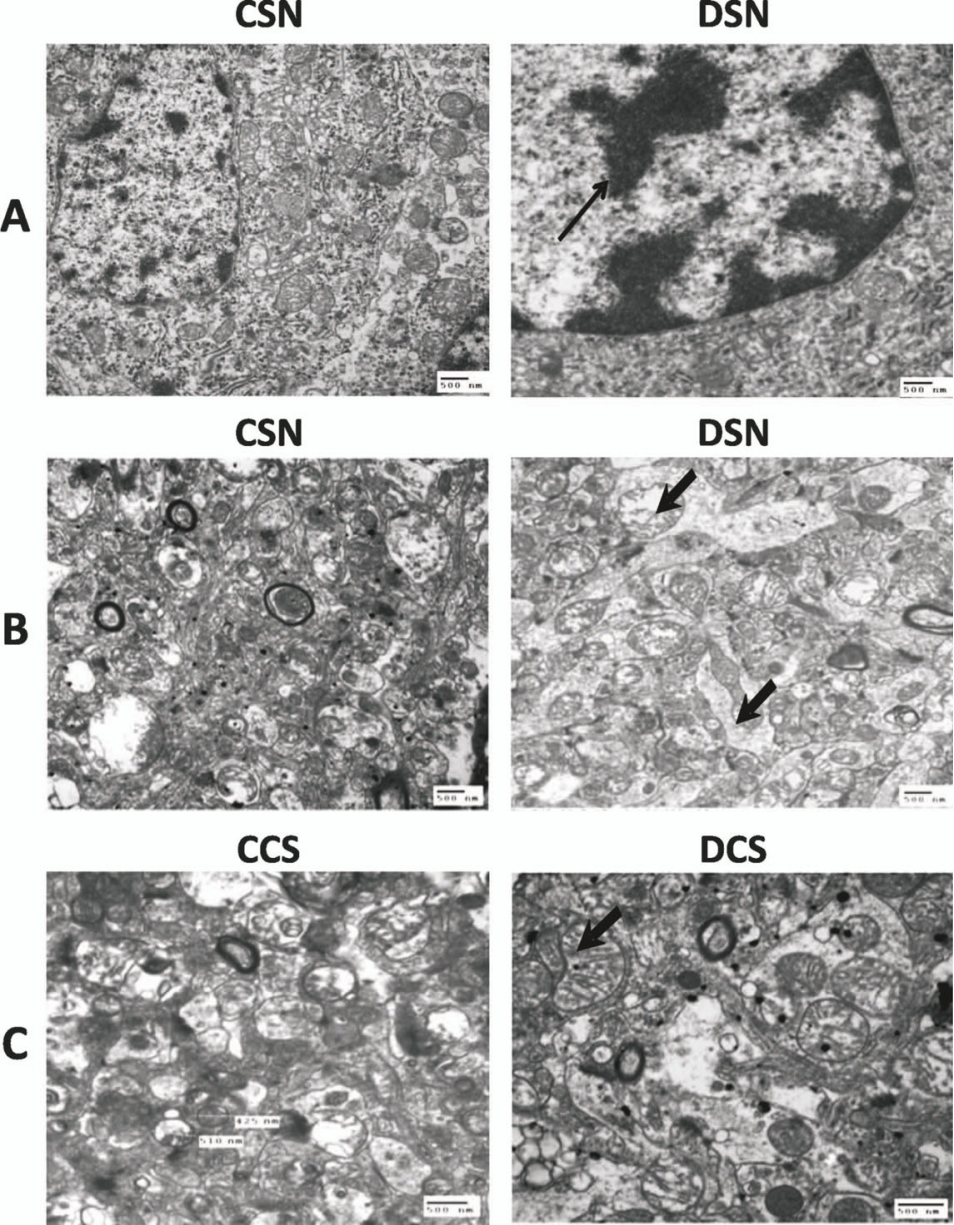
**Transmission electron micrograph of SN of rat brain regions showing the ultra structural changes of nucleus after chronic dichlorvos exposure**.**(A)**, Chromatin condensation and reduced cytoplasmic volume were observed in SN. Scale bars - 500 nm. **(B&C) **Relatively normal appearance of perinuclear cytoplasmic contents including mitochondria, endoplasmic reticulum and Golgi apparatus in control group. Dichlorvos exposed animals showed mitochondrial enlargement with more distorted cristae (thick arrow head) in the surrounding neuropil of neurons. CSN: substantia nigra; CCS: corpus striatum;DSN: Dichlorvos substantia nigra;DCS: Dichlorvos corpus striatum. Scale bar: 500 nm.

**Figure 4:**
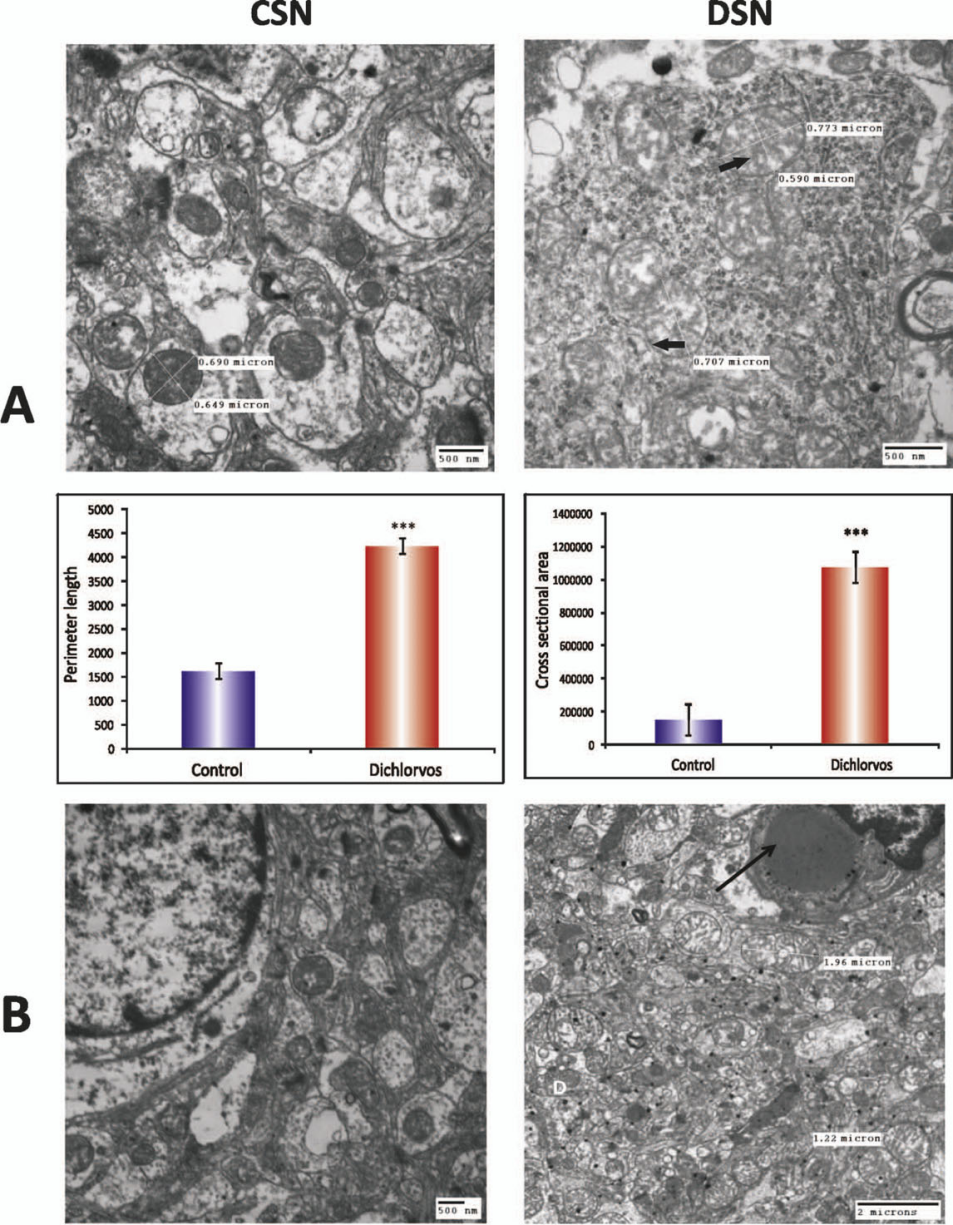
**(A) Analysis of mitochondrial perimeter length and cross-sectional area in the neuropil of the Substantia nigra**. Both the cross-sectional area/mitochondrion and perimeter length/mitochondrion for the dichlorvos group were significantly greater than the control group. (B) Ultra structure of SN neurons from dichlorvos treated rat brain containing perinuclear cytoplasmic inclusions. Scale bars: 500 nm (thin arrowhead perinuclear inclusions). CSN; Substantia nigra DSN: Dichlorvos Substantia nigra.

Electron microscopic studies were carried out in order to know if the biochemical changes observed after dichlorvos exposure were strong enough to leave an impact on the morphology of mitochondria from SN and CS from control and treated groups. Striking changes in the mitochondria of neurons and neuropil of the SN in dichlorvos exposed rats (Figures 3B, C, 4A) were observed. These changes were not observed in the control group. In the dichlorvos exposed rats, many mitochondria in the neuropil of the SN were greatly enlarged and swollen into spherical shapes rather than the normal ovoid or rod shape (Figures 3B, C, 4A, B). Frequently, these enlarged mitochondria had prominent distorted cristae (thick arrow 3B, C). To look at the differences in mitochondrial structure between two groups quantitatively, we systematically measured two mitochondrial parameters that reflected size and shape, the cross-sectional area and perimeter for each mitochondrion (Figure 4A) in a blinded fashion. The mean cross-sectional area per mitochondrion for the dichlorvos group was 1,079,363 nm^2^, compared to152, 302 nm^2^, controls. By T test and Mann-Whitney test the means were found to be significantly different (P < 0.001). The mean perimeter length per mitochondrion for the dichlorvos group was 4514.365 nm, compared to 1647.206 control group. T test analysis for mitochondrial perimeter length also resulted in identical increase as that for cross sectional area of mitochondrial (means were significantly different by t test at P < 0.001), (Figure 4A).

In the SN of dichlorvos treated animals, we found spherical inclusions in the perinuclear cytoplasm of a few neurons (Figure 4B). No aggregates or inclusions were found in control animal. In addition, chromatin condensation was also seen in the dichlorvos treated animals (Figure 3A). Many SN neurons in the dichlorvos treated group also appeared shrunken with reduced cytoplasmic volume and had nuclei with deep invaginations of the nuclear membrane (Figure 3A). Similar losses of cytoplasmic volume or invaginations of the nuclear membrane were not found in neurons from the control group.

### Effect of chronic dichlorvos exposure on Tyrosine hydroxylase activity in substantia nigra and corpus striatum of rat brain

Tyrosine hydroxylase is the rate limiting enzyme in catecholamine biosynthesis. It functions as an oxidoreductase and tetrahydrobiopterin acts as a co-factor to convert L-tyrosine to L-DOPA (L-dihydroxyphenylalanine). More over tyrosine hydroxylase is a marker for dopamine neurons. As is evident from Figure 2B, chronic administration of dichlorvos causes significant decrease in the TH enzyme activity in SN and CS as compared to control. Dichlorvos treated animals showed TH enzyme activity reduced to 52.33% in SN and 50.85% in CS compared to control animals. This suggests that dichlorvos could decrease dopamine levels by decreasing the tyrosine hydroxylase activity.

### Effect of chronic dichlorvos exposure on levels of Dopamine and its metabolites in SN and CS of rat brain

The impact of dichlorvos exposure on DA synthesis and storage was assessed in tissue samples from CS and SN. Chronic s.c injection of dichlorvos produced significant loss of dopamine in the SN and CS following 12 week of administration. The level of dopamine decreased by 73% in SN and 76.8% in CS respectively compared to control animals (Figure 2C). In addition to dopamine, it's metabolites levels were also significantly decreased. Dichlorvos treated animals showed 72.5% (SN) and 44.23% (CS) reduction in the levels of DOPAC compared to control animals (Figure 2D). In the case of HVA, dichlorvos treated animal showed 72.5% (SN) and 44% (CS) reduction when compared to controls (Figure 2E). These results indicate that dichlorvos administration may cause significant decrease in the levels of dopamine and its metabolites in SN and CS region of rat brain.

### Effect of chronic dichlorvos exposure on Dopamine -β- hydroxylase activity in substantia nigra and corpus striatum of rat brain

Dopamine-β-hydroxylase is a mixed function oxidase which catalyses the conversion of dopamine to norepinephrine and it is also a marker for noradrenergic neurons. No change in the activity of DBH was observed in any of the brain regions studied after chronic dichlorvos exposure. So it seems this dose of dichlorvos is not toxic to noradrenergic neurons but specifically affects dopaminergic neurons (Additional file 2).

### Effect of chronic dichlorvos exposure on Monoamine Oxidase A activity in substantia nigra and corpus striatum of rat brain

Monoamine oxidase is catecholamine degradative enzyme which deaminates dopamine and norepinephrine to their corresponding aldehydes. Monoamine oxidase A specifically deaminates dopamine. As shown in additional file 3, no significant change in the Monoamine oxidase enzyme activity was observed in any of the brain regions studied after chronic dichlorvos administration as compared to control. This indicates that chronic dichlorvos exposure is not affecting the degradative pathway. The decrease in the levels of dopamine may be due to the decreased activity of TH enzyme responsible for its synthesis.

### Effect of chronic dichlorvos exposure on the activity of acetylcholinesterase in rats

Acetylcholinesterase, which is the degradative enzyme of acetylcholine and thereby responsible for the termination of cholinergic response, exists as true (acetyl) cholinesterase and pseudo (butyryl) cholinesterase. The true cholinesterase is the functional enzyme of the cholinergic system and forms a major part of the total cholinesterase in brain. Following chronic (2.5 mg/kg b.wt./day for 12 weeks, sc) dichlorvos exposure it was observed that there was no significant decrease (Additional file 4) in the activity of brain acetyl cholinesterase in treated rats as compared to controls. So, at this dose regime cholinergic neurons are not affected.

#### General behavioral, motor impairments and catalepsy behavior

Having observed dopamine depletion after chronic dichlorvos administration in SN and CS of rat brain, it was imperative to assess motor functions, open field and catalepsy as these are known to be affected by dopamine levels.

#### Open field

Locomotor activity refers to the movement from one location to another. In rodents, one of the most important component of exploration, a prominent activity of the rat's repertoire of spontaneous activity, is locomotion. Moreover, locomotor activity and exploration are involved in many behavioral and physiological functions. The Open Field test is commonly used to assess locomotor behavior. Furthermore, locomotor abnormalities are associated with several human diseases such as Parkinson's and Huntington's disease or Hyperactivity syndrome and are also displayed by animal models. Hence, we studied the open field behavioral functions after chronic dichlorvos exposure.

**Figure 5:**
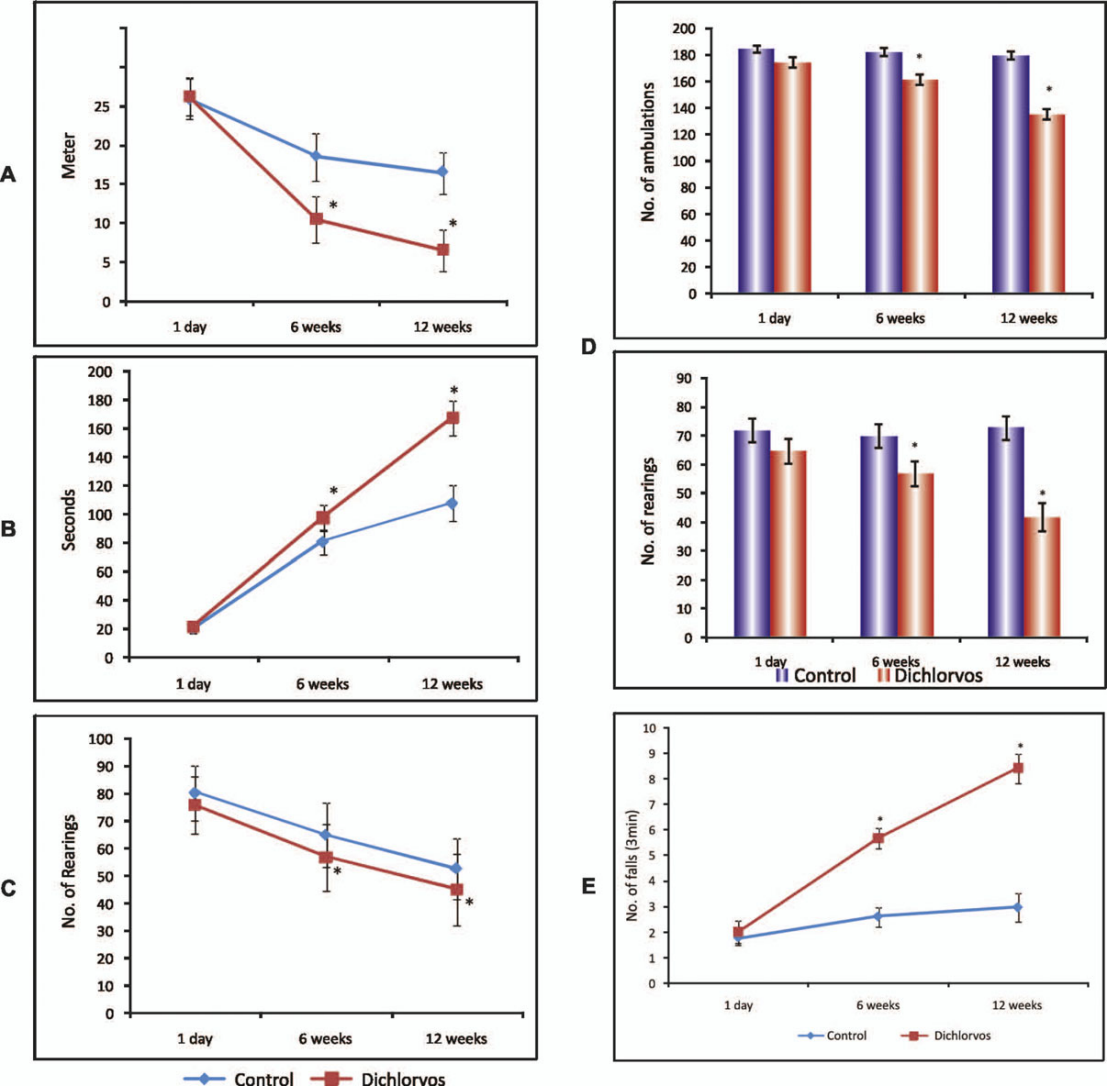
**Effect of chronic dichlorvos exposure on-Open field behaviour**. Locomotor activity. (B). Immobile time (C). No.of rearing. (D). Spontaneous locomotor activity (No. of amulations and No. of rearing). (E). Rota rod test. Dichlorvos treated rats received 2.5 mg/kg b.wt. of dichlorvos, s.c., for 12 weeks and control animals received equal volume of corn oil. The values are mean ± S.D. of 6 animals in each group. * p < 0.05 significantly different from control group.

Rats were tested for behavioral activity six weeks and twelve weeks after dichlorvos administration. Figure 5 illustrates the effect of chronic dichlorvos administration to rats on the locomotor activity in the open field. Six weeks after chronic dichlorvos administration, rats exhibited a decrease in locomotor activity which was more significant at 12 weeks of dichlorvos exposure. Not only locomotor activity, but the other components of open field like total distance travelled, immobile time and rearing were also affected at 6 and 12 weeks of post exposure and the alterations were more pronounced at 12 weeks post exposure when compared to control (Figure 5A-C).

Rearing and ambulation were also observed by Actophotometer. Again there was significant difference between control and treated animals as depicted in Figure 5D after12 weeks of exposure.

#### Motor function test

PD patients show increasing motor impairment, gait and postural difficulties and cognitive dysfunction. In addition to this, different PD animal models also show significant motor impairments. So, we were interested to know if the muscle strength and motor coordination was affected after chronic dichlorvos administration or not?

#### Rota rod performance

The rota rod test was carried out to assess the muscle strength and motor impairments of animals following chronic dichlorvos administration.

The rota rod test (Figure 5E) revealed a marked impairment in the muscle strength and coordination. There was a significant (p < 0.05) reduction in the retention time at 6 week and 12 week. The treated animals showed neuromuscular incoordination and seemed totally disorientated and confused during the training period as compared to control animal. None of the treated animals could maintain themselves on the rotating rod for the full quota of the cut off time (180 s).

#### Chronic dichlorvos administration and catalepsy

Catalepsy in laboratory animals is defined as a failure to correct an externally imposed posture. When a normal animal is placed in an unusual posture, it will change its position within seconds. A cataleptic animal, on the other hand, will maintain this posture for a prolonged period of time (i.e., several minutes or longer). Catalepsy was of interest to us because of its similarity to symptoms of such human disorders as Parkinsonism and brain damage involving parts of the basal ganglia.

Cataleptic scores of the present study are given in additional file 5a&b, assessed by block method and metal bar test, respectively. Dichlorvos administered animals showed significant increased cataleptic scores in block test. Bar test also showed the same result. Dichlorvos administration lead to progressive and significantl development of catalepsy (p < 0.01) at a dose of 2.5 mg/kg after 6^th ^week and 12^th ^week. However, there was no pronounced increase in the cataleptic scores in control animals after 12 weeks. The maximal increase (P < 0.01) in catalepsy behaviour was observed after 12 weeks in both tests. So, the catalepsy was developed gradually and it showed maximum impact after 12 weeks of dichlorvos administration.

### Dopaminergic neurodegenaration: expression of phenotypic markers (TH) and cytoplasmic bodies containing alpha synuclein and ubiquitin

#### **Nigrostriatal dopaminergic degeneration (**Immunohistochemistry for TH)

**Figure 6:**
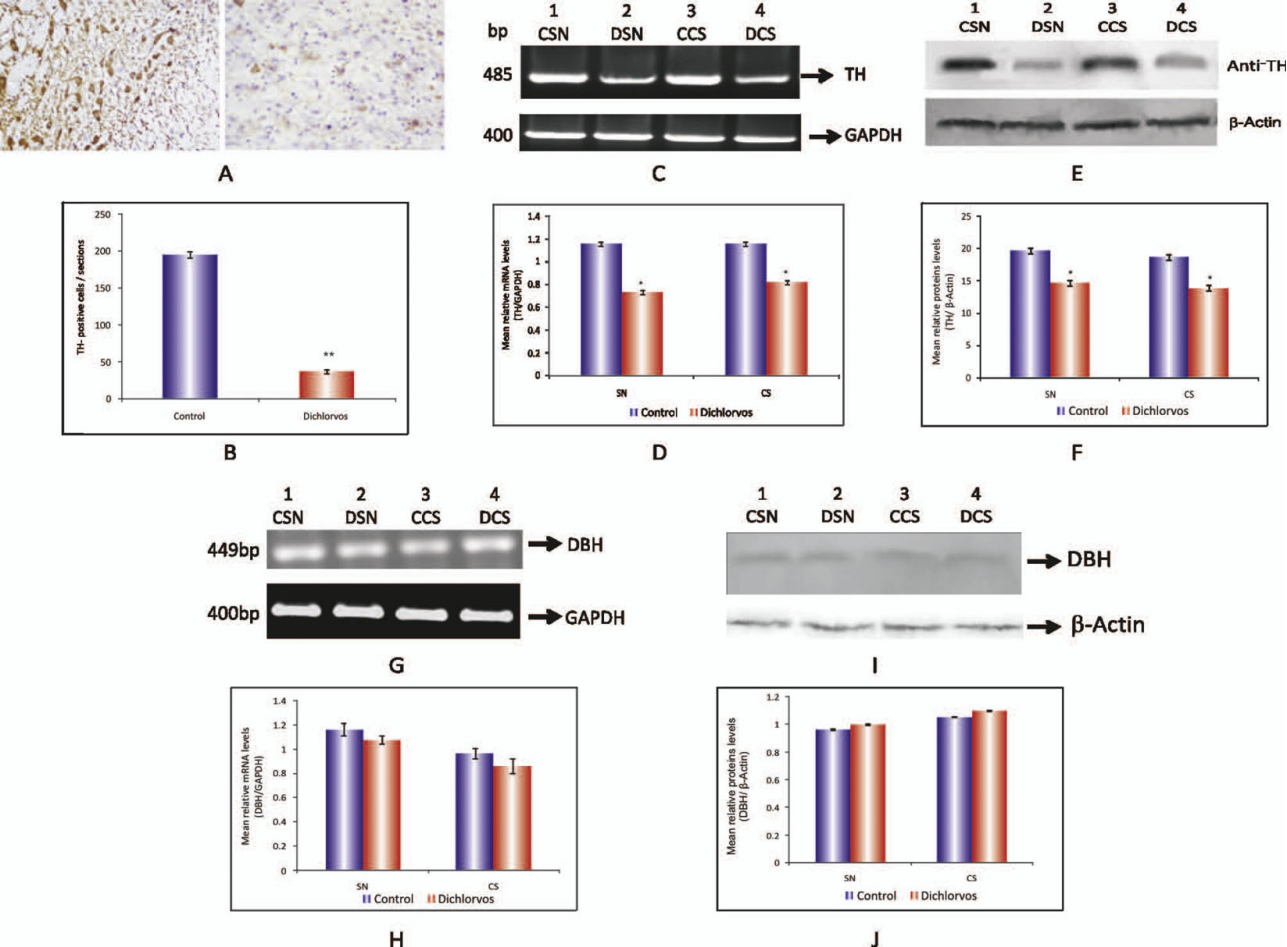
**Nigrostriatal dopaminergic degeneration**. (A) Representative image show TH expression in SN area of dichlorvos treated rat and control littermate. (B) Bar graph depicts TH-immunoreactive, Cell counts in the SN of dichlorvos rat and control littermate. Values are mean ± S.D. (N = 6),** p< 0.01 significantly different from control group. (C). Semi quantitative RT-PCR analysis of TH from substantia nigra and corpus striatum of rat brain. (D) Densitometric analysis of TH/GAPDH. (E). The proteins separated on 10% SDS-PAGE were subjected to immunoblotting using anti-TH antibody. (F) Densitometric analysis blot. TH/β actin. (G) Effect of chronic dichlorvos exposure on DBH mRNA expression in SN and CS by RT-PCR (Agarose gel electrophoresis).(H) Densitometric analysis of DBH/GAPDH. (I). Effect of chronic dichlorvos exposure on DBH quantitative protein expression in substantia nigra and corpus striatum of rat brain. The proteins separated on 12% SDS-PAGE were subjected to immunoblotting using anti-DBH. (J) Densitometric analysis blot DBH/β actin. Values are mean SD of 4 animals in each group, *p < 0.05 significantly different from control group. Lanes 1,3: Control; Lanes 2,4: Dichlorvos treated; CSN: Control substantia nigra; DSN: Dichlorvos substantia nigra; CCS: Control corpus striatum; DCS: Dichlorvos corpus striatum.

Immunohistochemistry of phenotypic marker of dopaminergic neurons, TH indicated dopaminergic neurodegeneration (Figure 6A). At 12 weeks of dichlorvos administration, the number of TH-positive neurons in the SN was reduced by 60-70% as compared to control rats (Figure 6B). We found that chronic dichlorvos administration caused nigral cell loss in rat brain, as documented by a large decrease in TH-positive neurons in the SNpc and TH-positive fibers in the CS and this was associated with a decline in the levels of dopamine and dopamine metabolites (DOPAC, HVA) by approximatly 75% (Figure 2C-E). In the next experiment RT-PCR was performed to confirm the above histochemical finding.

### Expression of tyrosine hydroxylase mRNA in SN and CS by RT-PCR

In order to confirm the decline in TH positive neurons (histochemistry result), it was imperative to study the expression of TH at transcription level by RT-PCR. We measured the expression of TH mRNA in SN and CS region after chronic dichlorvos administration. Densitometric analysis showed that dichlorvos caused significant decrease in TH mRNA levels in these regions as compared to control (Figure 6C, D).

### Quantitative protein expression of tyrosine hydroxylase

To confirm decrease in TH protein following decrease in TH-mRNA by dichlorvos exposure, TH protein level were examined. The TH protein level was examined (homogenate from SN and CS region) by Western blotting. Densitometric analysis of the bands revealed that (Figure 6E, F) dichlorvos causes significant decrease in TH protein expression compared to control. This result was similar to that of RT-PCR result which also demonstrated decreased expression of TH at transcription level.

### Effect of chronic dichlorvos exposure on DBH mRNA expression in SN and CS by RT-PCR

The results described above demonstrated that dichlorvos exposure affects TH expression at both the transcription as well as translational levels. Further, we studied the expression pattern of DBH. It was observed that chronic dichlorvos administration did not affect the activity of DBH enzyme. In order to further confirm these results, we studied the DBH mRNA level by RT-PCR after chronic dichlorvos administration. Densitometric analysis revealed that there was no significant change in the DBH mRNA levels in SN and CS of treated animals compared to controls (Figure 6G, H).

### Quantitative DBH protein expression in SN and CS of rat brain after chronic dichlorvos exposure

For the quantitative determination of DBH protein, western blot analysis was performed using specific antibody against DBH in the crude extract prepared from the SN and CS of control and dichlorvos treated animals. The crude preparation from SN and CS were probed with anti-DBH antibody. The microdensitometic analysis of this western blot showed that dichlorvos exposure did not show any significant change in the expression of DBH when compared to controls. Taken together protein expression, mRNA expression and enzyme activity of DBH, it is clear that at this dose of dichlorvos, it is not affecting the adrenergic neurons (Figure 6I, J).

### H&E staining and expression of neurofilametary protein in cortex after chronic dichlorvos exposure

**Figure 7:**
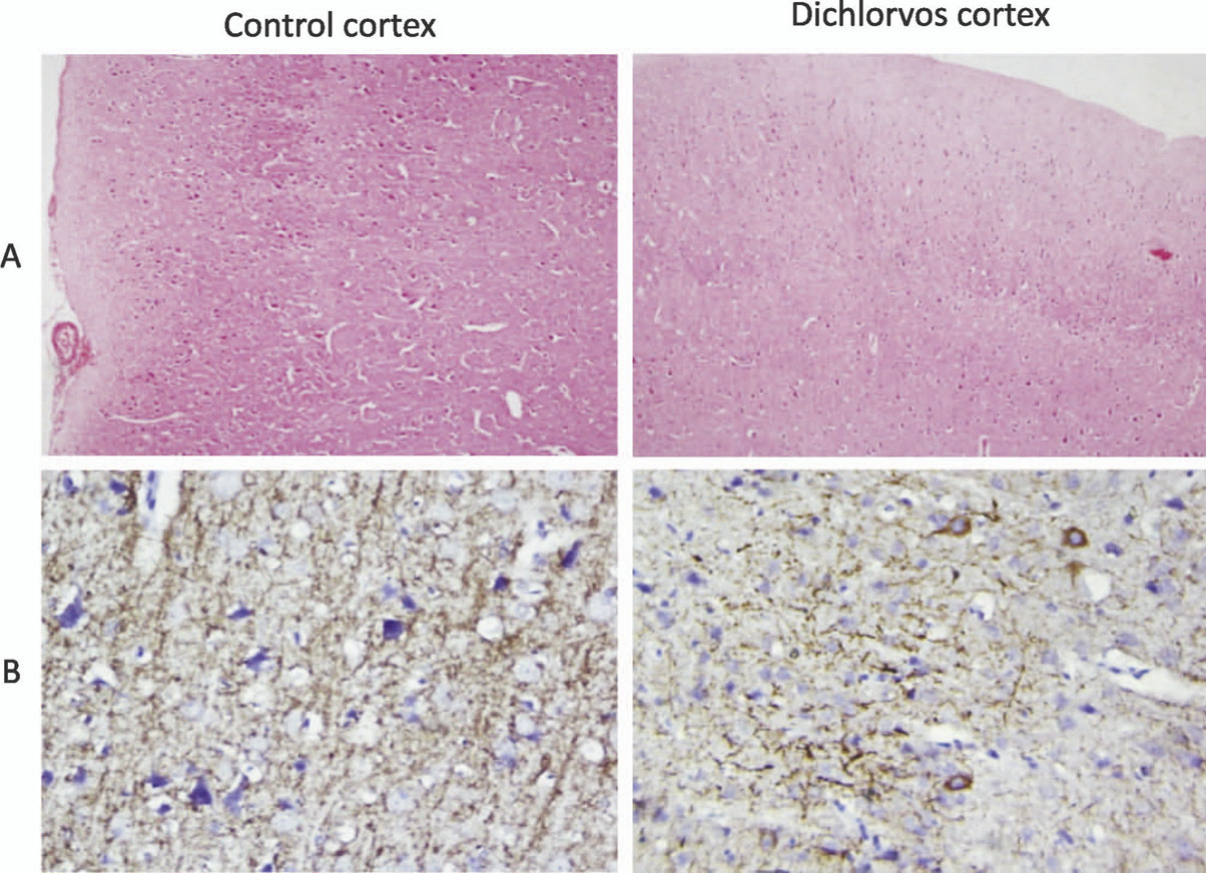
**No neuronal loss occurred in the cortex of dichlorvos exposed animals**. (A) Representative H&E image show healthy neuron in cortex area of dichlorvos treated rat and control littermate. (B) Representative neurofilament protein image show no neuronal loss in cortex area of dichlorvos treated rat and control littermate.

In order to confirm, whether this dose regime of dichlorvos exposure is affecting other neurons than dopaminergic neurons, cortical region of control and treated sections were stained with H&E and neuron specific neurofilament protein (NFP). These results showed that, all cortical neurons are intact and there was no neurodegeneration in cortical region (Figs 7A, B). So, at this dose it was specifically affecting the dopaminergic neuron.

### α-synuclein and ubiquitin protein aggregates in substantia nigra after chronic dichlorvos exposure

**Figure 8:**
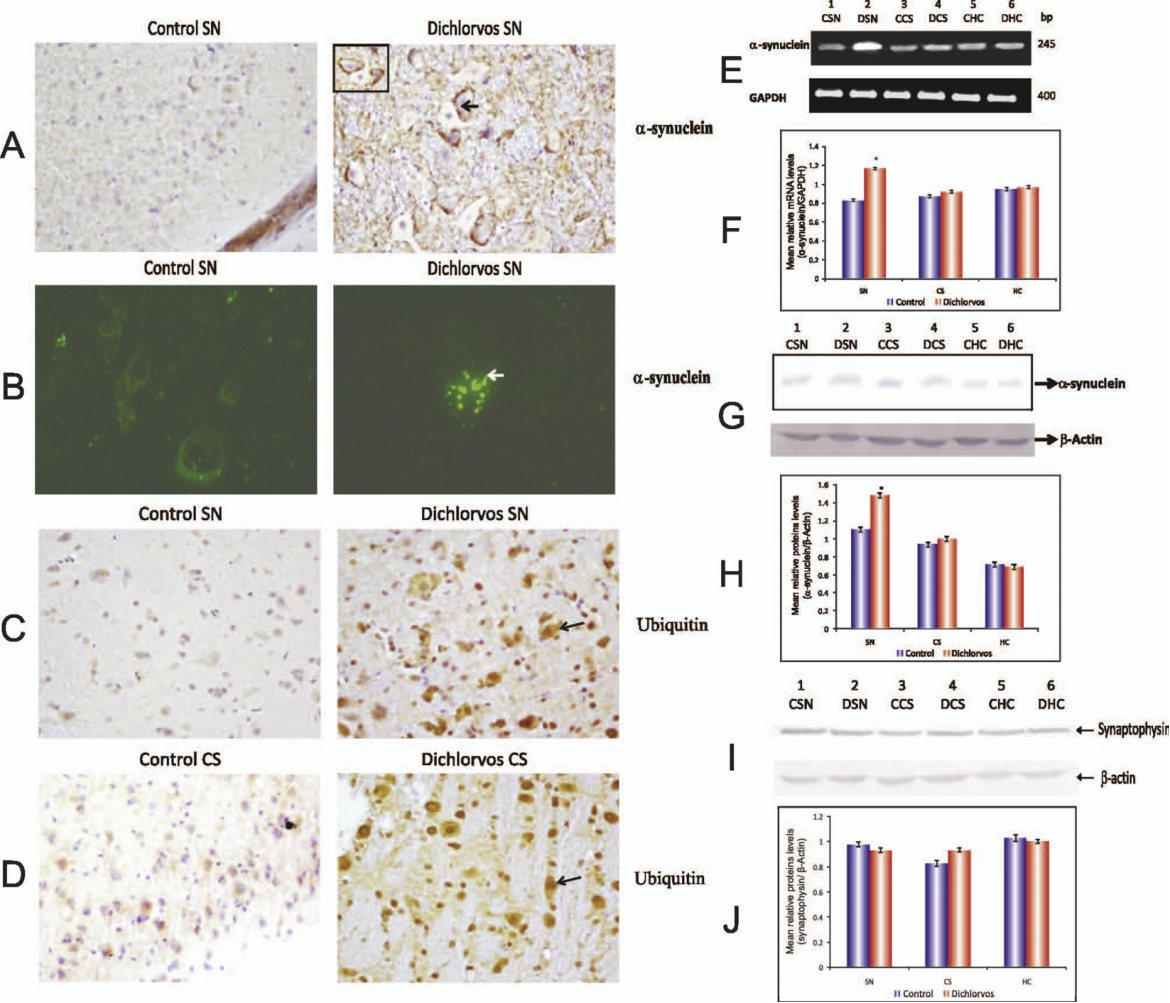
**α-synuclein and ubiquitin protein aggregates in SN after chronic dichlorvos exposure**. (A) The brain slides were probed with anti α -synuclein and ubiquitin antibody. The signals were revealed using HRP conjugated secondary antibody and photographed.(B)The brain slides were probed with anti α -synuclein and ubiquitin antibody. The signals were revealed using FITC conjugated secondary antibody and photographed. (C and D) The brain slides were probed with anti ubiquitin antibody. The signals were revealed using HRP conjugated secondary antibody and photographed. (E). Effect of chronic dichlorvos exposure on α -synuclein mRNA expression in SN and CS by RT-PCR.(F) Densitometric analysis α-synuclein/GAPDH.(G) Effect of chronic dichlorvos exposure on α-synuclein quantitative protein expression in SN and CS of rat brain. The proteins separated on 10% SDS-PAGE were subjected to immunoblotting using anti α-synuclein antibody. (H). Densitometric analysis blot α-synuclein/β actin. (I) Effect of chronic dichlorvos exposure on synaptophysin quantitative protein expression in substantia nigra and corpus striatum of rat brain. The proteins separated on 10% SDS-PAGE were subjected to immunoblotting using anti-synaptophysin antibody. (J) Densitometric analysis blot synaptophysin/β actin. Values are mean SD of 4 animals in each group, *p < 0.05 significantly different from control group. Lanes 1,3,5: Control; Lanes 2,4,6: Dichlorvos treated; CSN: control substantia nigra; DSN: Dichlorvos substantia nigra; CCS: Control corpus striatum; DCS: Dichlorvos corpus striatum; CHC: Control hippocampus; DHC: Dichlorvos hippocampus.

Nigrostraital neuronal death, cytoplasmic protein aggregates, known as Lewy bodies, containing α-synuclein and ubiquitin are pathologic hallmarks of PD [15]. Here, we sought to determine whether there were inclusion bodies in the SN by staining sections with antibodies against ubiquitin and α -synuclein. It was observed that many nigral cells in dichlorvos treated animals demonstrated α-synuclein positive aggregates along with higher amount of ubiquitin (Figure 8A-D). Immunofluorescence staining was done to confirm the cytoplasmic inclusions in the cytoplasm of nigral neurons (Figure 8B). No aggregation was seen in the SN region of control animals. Moreover, this aggregation was specifically seen only in the SN region of treated animal, as we were not able to find any aggregation in the hippocampus and CS. The Lewy bodies seen in PD brains contain ubiquitin in an insoluble form, and chronic *in vivo *rotenone exposure also reproduced these ubiquitin-positive inclusions [3]. Therefore, in order to see if dichlorvos exposure also caused inclusions of ubiquitin, we estimated ubiquitin in the control and dichlorvos exposed rats. Immunohistochemistry of treated rat brains revealed a significant increase in cytoplasmic ubiquitin staining in SN and CS region (Figure 8C, D).

### Up-regulation of α-synuclein mRNA following dichlorvos administration

To determine whether changes in α-synuclein protein expression are accompanied by changes in α-synuclein mRNA levels, SN samples from chronic dichlorvos-treated rat brain were used for semiquantitative RT-PCR amplification. Other regions, such as the CS and the hippocampus, were also analyzed. Consistent with α-synuclein protein alterations, α-synuclein mRNA expression was increased in the SN following chronic dichlorvos exposure. No changes in α-synuclein mRNA expression were found in either the CS or the hippocampus of treated animals (Figure 8E and 8F).

### α-synuclein and synaptophysin protein expression after chronic dichlorvos exposure

To determine whether α-synuclein may be involved in the deleterious cascade of events induced by dichlorvos, we also assessed α-synuclein protein expression levels in the SN, CS and hippocampus of dichlorvos-treated rat brain. Western blot analysis revealed single band of 19 kDa α-synuclein. After chronic dichlorvos exposure, α**-**synuclein protein expression significantly increased only in SN extracts (Figure 8G, H). No change in α-synuclein protein level was detected in the CS or in other regions, such as hippocampus (Figure 8G, H). In addition to α-synuclein, levels of synaptophysin, another presynaptic protein were also estimated so as to ascertain the possibility that α**-**synuclein alterations might not be a part of nonspecific response of synaptic-related proteins to dichlorvos injury. In contrast to changes in α-synuclein protein levels, β actin and synaptophysin protein levels remained unchanged in the SN after chronic dichlorvos administration (Figure 8I, J).

## Discussion

Epidemiological studies have repeatedly indicated that pesticide exposure is a significant risk factor for PD. However, mechanistic link between increased risk for PD and chronic exposure to pesticides remains to be established. Several reports demonstrate modest but reproducible reductions in mitochondrial complex I function in a variety of tissues from PD patients, including brain, platelets, muscle and fibroblasts. This finding suggests a systemic complex I defect in PD [3,16,17]. The role of mitochondria in PD has been further accentuated by the observation that MPP+, the active metabolite of 1-methyl-4-phenyl-1,2,3,6- tetrahydropyridine (MPTP) and an inhibitor of complex I of the mitochondrial electron transport chain (ETC) causes an acute parkinsonian syndrome [18]. Inhibition of complex I blocks the flow of electrons along the mitochondrial electron transport chain, which results in increased production of ROS. We also observed mitochondrial complex 1 along with complex 1V inhibition accompanied by increased levels of ROS production after chronic dichlorvos treatment.

Evidence for the involvement of oxidative stress in PD has also been obtained from PD patients. Increased lipid peroxidation and oxidative damage to DNA and proteins have been observed in SN of PD patients [19]. We also observed increased lipid peroxidation and increased ROS levels in SN and CS of chronic dichlorvos treated animals. Oxidative damage in PD may not be selective to SN as elevated oxidative protein damage has also been reported throughout PD brain. Animal models of PD have also suggested the involvement of oxidative stress. MPTP-treated mice and dieldrin treated N27 cells demonstrate elevated levels of ROS and lipid peroxidation [20-22]. These results are in agreement with our results. Furthermore, the *in vitro *rotenone model of PD has also suggested the involvement of oxidative stress in neurodegenaration [23]. Our results indicate that dichlorvos treatment (*in vivo*) produces marked mitochondrial and neuritic pathology in the SN. Control animals did not produce pathologic changes in the SN and CS. The mitochondrial changes found in this study most likely reflect the sequelae of injury to mitochondria, but in addition or alternatively could indicate the involvement of mitochondria in producing neuronal pathology. Changes seen in the dichlorvos treated animals are similar to abnormalities described in PD and MPTP-treated animal models. Mitochondria from patients with PD have been described as swollen with rounded rather than rod-like profiles, to have loss of cristae and to often have discontinuous outer membranes [24]. Studies in MPTP-treated animal models have also reported enlarged mitochondria with disordered mitochondrial cristae [25]. These finding are strikingly similar to what we have observed in the SN of our chronic dichlorvos exposed rats. As dendritic mitochondria tend to be larger than axonal mitochondria, (noted during our qualitative comparisons for structural changes), a greater impact on dendritic mitochondria may also explain the change in distribution of measurements for the dichlorvos group relative to the control group that suggests greater effect on larger mitochondria rather than proportionate increase equally affecting the smallest mitochondria. As our mitochondrial measurements were done on all the mitochondria on each captured EM field of view, the quantitative changes in the dichlorvos group may also or instead reflect greater mitochondrial effects on dopaminergic neurites independent of whether dendritic or axonal. Dendrites have been demonstrated to be able to initiate and spread apoptotic cascades to the neuronal cell body [26] which may form the basis of potential mechanisms of neurodegeneration. Our findings of, swollen and disintegrating mitochondria, autophagic vacuoles and cytoplasmic shrinkage in dichlorvos exposed animal SN have all been also observed in the processes of neuronal cell death and in the SN in PD [27]. Further support of dichlorvos affecting vulnerability to mitochondrial toxins, the pesticide paraquat, and rotenone can inhibit mitochondrial complex I activity and produce a PD-like syndrome [3,28].

We observed significant decrease in the dopamine levels in SN and CS after chronic dichlorvos administration (Figure 2C-E). Keeping in view the significant dopaminergic neuronal death we were interested to study in detail the dopamine metabolism viz synthetic as well degradative enzymes leading to altered levels of dopamine after dichlorvos exposure. Although we observed no change in enzyme activity, protein expression as well as mRNA level for DBH and Monoamine oxidase A, after chronic dichlorvos exposure (Figure 6G-J, additional file 2, 3). But at the same time TH activity was significantly decreased after dichlorvos exposure (Figure 2B). Our immunohistochemistry results of TH-positive neuron counts showed that the number of TH positive neurons decreased by 84.70 ± 3.4% in the SN of the dichlorvos exposed animals (Figure 6A and 6B), compared to that in the control group. Likewise, the Western blot and RT-PCR results showed there was a reduction of TH mRNA and protein expression in the SN and CS of the dichlorvos treated animals (Figure 6C-F). These result confirmed that dichlorvos is acting at dopamine synthesis stage and not at degradative pathway. This toxic effect was highly specific to dopaminergic neurons, because cortical neuronal are not affect (Figure 7). This is also in agreement with findings of Betarbet et al [3] that systemic rotenone infusion resulted in nigrostriatal dopaminergic degeneration and decreased dopamine levels.

It is important to mention that changes in α-synuclein expression and aggregation after dichlorvos exposure are region specific because α-synuclein mRNA and protein expression was increased only in SN and not in other regions, such as hippocampus and CS. On the other hand, increased α-synuclein expression does not seem to be part of a common response of synaptic related proteins to dichlorvos injury because the expression levels of synaptophysin, another synapse-associated protein, were either unchanged or the changes were insignificant after chronic dichlorvos exposure. The observation that levels of α-synuclein mRNA is also increased rather supports the view that following dichlorvos administration α-synuclein is up-regulated and accumulates in the SN. Altogether, our data raise the possibility that α-synuclein up-regulation, which occurs in specific context to dichlorvos-induced apoptotic death in SN dopaminergic neurons, contributes to the cascade of deleterious events that ultimately kill these cells. Up-regulation of α-synuclein may alter the normal intracellular trafficking of certain proteins that, like many of the Bcl-2 family members, depend on being at a specific intracellular location to exert their regulatory effects on apoptosis [29,30].

There is also compelling evidence to indicate that α-synuclein has a significant propensity to aggregate, and that this property can be enhanced by the familial PD-linked mutations or by posttranslational modifications, such as produced by oxidative stress [30-32]. Relevant to this is the fact that α-synuclein is present in high amounts in the inclusion LB, which is regarded by some as a key factor in the demise of SNpc dopaminergic neurons in PD. In the dichlorvos exposed rat, we identify the formation of inclusions in SN region. This leads to the possibility that α-synuclein up-regulation may play a role in the dichlorvos-induced neurotoxic process, through the formation of LB-like inclusions.

We have also found that mitochondrial dysfunctions by dichlorvos exposure also stimulate production of reactive oxygen species. Moreover, we have previously reported that 6 mg/kg of dichlorvos exposure over a period of 12 weeks produces, oxidative damage to proteins and DNA, and sensitizes cells to subsequent oxidative stressors; eventually, leading to release of cytochrome c from mitochondria to the cytoplasm [11]. This mechanism could also explain the cytoplasmic inclusions found in nigral neurons of dichlorvos treated rats, because both oxidative damage and cytochrome c enhance α-synuclein aggregation. The recent discovery that an increased level of the α-synuclein gene resulting from the triplication of α -synuclein locus causes PD in some individuals and mutant α-synuclein selectively expressed in astrocyes, induced rapid progressed paralysis in mice. These data strongly suggests that over expression and mutation of this gene could be a risk factor for PD [33,34]. The α-synuclein over expression due to exposure to environmental pesticides is likely to influence increased vulnerability of nigral dopaminergic neurons.

He-Jin Lee et al [35] provided the first cell-based evidence that mitochondrial dysfunction may result in α-syn aggregation. One of the outcomes of ETC inhibition is an increased production of free radicals, hence increased oxidative stress. In recent studies, several groups have shown that the aggregation of α- synuclein could be promoted by oxidative and nitrative stresses [36,37]. Indeed, oxidizing and nitrating agents induced dityrosine cross-linking of recombinant α-synuclein and stabilized preassembled aggregates [38]. Furthermore, accumulation of nitrated α- synuclein was demonstrated in the inclusions of PD, dementia with Lewy bodies, Lewy body variant of Alzheimer's disease and multiple system atrophy, implicating the role of oxidative stress in LB formation in α-synucleinopathies [39]. We also observed ETC inhibition, increased ROS production and oxidative stress after chronic dichlorvos administration and this may be one of the pathway leading to aggregation of α- synuclein after dichlorvos exposure. Another outcome of mitochondrial dysfunction is a defect in energy production (ATP generation). He-Jin Lee [35] also showed tight temporal correlations between ATP level and α- synuclein aggregation both in depletion and recovery phases, suggesting that an impaired energy supply may also play a role in α- synuclein aggregation. This also supports our study, as we observed decreased ATP production after chronic dichlorvos administration.

Protein aggregation is considered to be a manifestation of a disturbed cellular protein-folding homeostasis, which is maintained by at least two defense mechanisms against damaged (misfolded) proteins: degradation by the ubiquitin-proteasome (Ub-Pr) system and the chaperone-mediated refolding system. Impairment of these systems, most of which are dependent on ATP, will cause accumulation of the misfolded proteins. Therefore, while oxidative stress can increase the rate of protein misfolding, the concomitant reduction in ATP levels can decrease the rate at which the cells rescue or remove the misfolded proteins, thus resulting in protein aggregates. Interestingly, unlike globular proteins, α-syn in isolation does not appear to have any stable structure [40]. To initiate the aggregation process, α-syn has to undergo a structural transition to form an aggregation-prone, partially folded intermediate, which is equivalent to the misfolded proteins in the aggregation process of globular proteins. In fact, the presence of the partially folded intermediate and the stabilization of this conformation in the α-syn aggregation process were demonstrated in recent studies [41,42]. Furthermore, the degradation of α- synuclein is mediated by the ubiquitin-proteasome system [43,44]. These results suggest that the aggregation of α- synuclein might be under control of the same defense mechanism as globular proteins. Therefore, the correlation between the ATP level and α- synuclein aggregation shown in our study suggest that the reduction in energy production, leading to impaired defense mechanism against misfolded proteins, is likely to contribute to the aggregation of α- synuclein.

All neurochemical as well as neuropathological changes seen after dichlorvos exposure seem to be reflected in the motor functions and catalepsy behavior of animals. Our results show that dichlorvos treated rats exhibited a significant reduction in locomotor activity and rearing frequencies observed in an open field at six weeks and twelve weeks of exposure. The dichlorvos exposed animals exhibited significant changes in motor activity and catalepsy (Figure 5, additional file 5a&b). This hypokinesia is an important feature of PD and it is assumed the same might have been produced after dichlorvos exposure. This result suggests that the lesion probably produced by a significant destruction of dopaminergic neurons of SN (Figure 6A) and consequent decrease in striatal dopamine levels may cause these symptoms to appear in our animals. Our study confirms that dopamine neuronal degeneration leads to decreased dopamine levels in the SN and CS of dichlorvos exposed animals and finally culminate in neurobehavioral impairments

## Conclusions

0ur study confirms dopamine neuronal degeneration and decreased dopamine levels in the SN and CS of dichlorvos exposed animals. Inspite of ban on dichlorvos usage by The United States Environmental Protection Agency in 1981, it continues to be available. This study high lights that dichlorvos exposure of a low dose and long duration may be toxic to DA neurons in rats. Taken together, our results indicate that chronic dichlorvos exposure may cause nigrostriatal neurodegenaration and renewing vents.

## Materials and methods

### Animals and their care

Male albino Wistar rats (150-200 g) were procured from the institute animal house and kept in well ventilated rooms in a 12-h light-dark cycle. Animals were provided standard rat pellet diet (Hindustan lever Ltd; Mumbai, India) and water ad libitum. Ethical clearance for killing of animals was duly obtained from Institute's Animal Ethics Committee. The animals were divided into following two groups (6 animals in each group), and four set of such groups were included in the study, (40 animals in total were used in whole study).

#### Control group

Animals received an equal volume of corn oil (vehicle) as administered to the animals of dichlorvos treated group.

#### Dichlorvos treated group

Animals received 2.50 mg/kg b.wt./day dichlorvos dissolved in corn oil, s.c. for 12 weeks. Three month exposure of rats to dichlorvos is equivalent to almost seven and half years of human exposure. At this dose regime acetylcholine esterase activity is not inhibited.

After the completion of treatment, animals were fasted overnight and sacrificed by decapitation using sodium pentathol. The brains were removed, rinsed in ice cold physiological saline (0.9% NaCl) and dissected into following regions: corpus striatum (CS), cortex, hippocampus and substantia nigra (SN) (as per the guidelines of Paxinos and Watson [45].

Mitochondria from rat brain regions were isolated by the method of Berman and Hastings [46].

### Mitochondrial NADH dehydrogenase (complexes I) activity

Complexes 1 activity was measured spectrophotometrically by the method of King and Howard [47] as described previously by Kaur et al [11]. This method involves catalytic oxidation of NADH to NAD+ with subsequent reduction of cytochrome c. The reaction mixture contained 0.2 M glycyl glycine buffer pH 8.5, 6 mM NADH in 2 mM glycyl glycine buffer and 10.5 mM cytochrome c. The reactionwas initiated by addition of requisite amount of solubilized mitochondrial sample and followed change in absorbance at 550 nm for 2 min.

### Mitochondrial cytochrome oxidase (complex IV) activity)

Cytochrome oxidase activity was assayed in brain mitochondria according to the method of Sottocassa et al [48] described previously by Kaur et al [11]. The assay mixture contained 0.3 mM reduced cytochrome C in 75 mM phosphate buffer. The reaction was started by the addition of solubilized mitochondrial sample and change in absorbance was recorded at 550 nm for 2 min.

### ROS generation

Mitochondrial ROS generation was assessed as described previously by Beretta et al [49] with slight modifications. Briefly, mitochondria were added to respiration buffer containing 5 mM pyruvate, 2.5 mM malate and 10 μM of dichlorodihydrofluorescein diacetate (H_2_DCFDA). Fluorescence was quantified after 20 min incubation using a Cary Eclipse fluorimeter (Varian, Palo Alto, USA) (excitation 488 nm, emission 525 nm).

### Biochemical markers of oxidative stress

#### Lipid peroxidation

It was assayed by the method of Wills [50]. Mitochondrial sample (0.5 ml) was diluted to 1.0 ml using Tris-HCl buffer (0.1 M, pH 7.4). The reaction mixture was incubated at 37°C for 2 h with constant shaking. At the end of the incubation 1.0 ml of TCA (10%, w/v) was added and then after thorough mixing the reaction mixture was centrifuged at 500 rpm for 10 min. To 1.5 ml supernatant, 1.5 ml TBA (0.67% w/v) was added and colour developed by placing the tubes at 100°C for 10 min in a boiling water bath. Samples were cooled and diluted with 1.0 ml DDW. The results were expressed as nmol MDA/μg protein.

### Antioxidant enzyme assays

#### Mitochondrial Superoxide Dismutase Assay (SOD)

Mitochondrial superoxide dismutase activity was measured by the method of MacMillan-Crow et al [51]. Mn-SOD activity in total solubilized mitochondrial extract was measured by the cytochrome C reduction method in the presence of 1 mM potassium cyanide to inhibit both Cu-Zn SOD and extra cellular SOD. The amount of enzyme required to produce 50% inhibition was considered as 1 U of Mn-SOD activity and results were expressed as U/mg protein.

#### ATP levels

ATP was determined luminometrically using ATP Bioluminescence assay kit (Sigma, St. Louis, MO, USA) according to the provided protocol. Mitochondrial samples were assayed for ATP content using the ATP dependence of the light emitting luciferase-catalyzed oxidation of luciferin. ATP nanomoles were calculated using a standard curve and related to protein content assessed using the method of Lowry.

### Estimation of dopamine and its metabolites

The levels of dopamine, 3,4- dihydroxyphenylacetic acid (DOPAC) and Homovanillic acid (HVA) were measured by high performance liquid chromatography (HPLC) with electrochemical detector (ECD) by the method of Gayle et al [52]. Waters^® ^standard system consisting of a high pressure isocratic pump, a 20 μl sample injector valve, C18 reverse phase column and electrochemical detector were used. Data were recorded and analyzed with the help of Empower software. Mobile phase consisting of 0.15 M NaH_2_PO_4_, 0.25 mM EDTA, 1.75 mM 1-octane sulfonic acid, 2% isopropanol and 4% methanol (pH 4.8). Electrochemical conditions for the experiment were +0.800 V, sensitivity ranges from 1 to 100 nA. Separation was carried out at a flow rate of 1 ml/min. Samples (20 μl) were injected manually. Brain sections (SN&CS) were homogenized (20%w/v) in homogenizing solution containing 0.1 M perchloric acid. After that samples were centrifuged at 24,000 × g for 15 min. The supernatant was further filtered through 0.25 μm nylon filters before injecting in the HPLC injection pump. Data were recorded and analyzed with the help of Empower^® ^software provided by Waters®. Concentration of DA, DOPAC and HVA were expressed as ng/mg was in wet tissue.

### Acetylcholinesterase assay

Acetylcholinesterase (AChE) activity was assayed in the brain homogenates(SN&CS sections) by the method of Ellman et al [53] wherein the hydrolysis of acetylthiocholine to thiocholine and acetate is measured. The thiocholine reacts with 5,5'-dithiobisnitrobenzoic acid (DTNB) to give a mixed disulfide and 5-mercapto-2-nitrobenzoic acid, a yellow compound which was measured spectrophotometrically at 412 nm.

### Tyrosine hydroxylase activity

Tyrosine hydroxylase activity was estimated by the method of Craine et al [54] and Shiman et al [55]. In a 2.93 ml reaction mix, the final concentrations are 57 mM Tris, 0.3 mM L-tyrosine, 0.2 mM 6,7-dimethyl- 5,6,7,8-tetrahyropterine, 0.15 mM ß-nicotinamide adenine dinucleotide, reduced form, 500 units catalase and 0.5 unit dihydropteridine reductase. 1.0 nanomole of L-DOPA from tyrosine per minute at pH 7.0 at 37°C. (one unit).

### Dopamine beta hydroxylase (DBH)

The activity of Dopamine β Hydroxylase (DBH) was assayed by the method of Kato et al [56] which involves the use of tyramine as the substract. Tyramine is converted to para- hydroxybenzaldehyde, which is isolated by successive ether extractions followed by ammonia and the difference between the absorbance at 333 and 360 nm corresponds the DBH activity.

### Monoamine oxidase B activity

Monoamine oxidase activity was measured by the method of McEwen and Cohen [57]. Benzaldehyde production from the oxidative deamination of benzylamine allows a convenient spectrophotometer assay of the enzyme activity.

### Electron microscopy

The dissected brain sections were fixed with 3% glutaraldehyde (diluted in 0.2 M Sorenson's buffer for) for 24 h at 4°C. The fixed brain sections were cut into approximately 1 mm cubes. The cubes were post fixed in 1% OsO4 for 2 h at 4°C and then dehydrated in an ethanol series and teated with propylene oxide for 10 min(two changes) at room temperature. The tissues were infiltrated with EPON mixture and propylene oxide (1:1) for 2 hr at room temperature and then embedded in EPON mixture containing Taab/812, followed by polymerization at 60°C for 24 hr. 0.5 μm thick sections were cut using ultra microtome (Reichert-Jung) and stained with 0.5% toluidine blue to confirm the presence of neurons. Then the 60 nm ultra thin sections were cut and mounted on Nickel grids (300 mesh). The sections were double stained with uranyl acetate and lead citrate and then examined by Transmission Electron Microscope (ZEISS 906, Germany) and photographed.

### Morphological analysis of Mitochondria

Three randomly acquired EM images of the neuropil from each brain region of interest (SN& CS) from each animal were photographed, digitally scanned at 600 dots per inch, and then alphanumerically coded for blinded analysis. All distinct mitochondria from each image were outlined by a single trained technician using Scion Image for Windows to obtain measurements of cross-sectional area and perimeter for each mitochondrion. Mitochondria were identified by the presence of both a distinct double membrane and identifiable cristae. Organelles that could not be clearly identified as mitochondria were not measured.

### Immunohistochemistry of Tyrosine hydroxylase (TH), NFP and α- synculin

After sacrificing the rat, brain regions (SN, CS and cortex) were isolated and washed with normal saline followed by 50 ml of 4% paraformaldehyde in phosphate-buffered saline (PBS), postfixed in 10% formalin for 5 days, and then paraffin embedded. Paraffin sections stained with haematoxylin and eosin were examined by light microscopy. For immunohistochemistry brain sections (4-5 microns) were cut on poly-L-lysine coated clean slides. For SN, sections were cut through the entire SN on a microtome. Every fourth section through the rostral-caudal extent of the SN was stained with an antibody against tyrosine hydroxylase. The sections were fixed overnight at 56°C. To deparaffinize, the slides were put in oven at 60°C for 2-3 min to melt the wax and were then put in xylene for 15 min followed by absolute alcohol for 5 min, 90% alcohol for 5 min, 50% alcohol for 5 min and finally in distilled water for 5 min. The slides were dipped in freshly prepared blocking solution of 1% H_2_O_2 _in methanol for 20 min in order to quench endogenous peroxidase. The washings were given three times in PBS for 5 min each. The slides were incubated in respective primary antibodies (anti TH, 1:100; NFP,1:100/α- syn, 1:150 (santa cruz USA) overnight at 4°C and washings were given three times in PBS for 5 min each. After washing, sections were incubated with HRP labeled secondary antibody (1:300) for 40 min. Sections were again washed three times with PBS and covered with 3,3'-diaminobenzidine (DAB) solution for 10 min at room temperature followed by washing in distilled water. Sections were then counterstained with haematoxylin and mounted with DPX. Cortex tissue sections stained by hematoxylin and eosin

### Cell count analysis

For quantitative analysis of the TH-positive neurons in the SN, the midbrain from −4.75 to −5.05 mm anterior to bregma was cut and the coronal sections were mounted serially on 5 slides, with 10 sections and 30 μm intervals between the adjacent sections in each slide. Among the 5 slides, the adjacent 2 slides were stained for TH. The counts of TH-positive neurons in SN were performed at a magnification of 20X in each section on both treated and control animals. Cell numbers are expressed as the mean number per section from these 10 sections. For quantitative analysis of the number of SN neurons, conventional procedures were performed. Neuron density was measured and averaged from four to six points of equal site, around which cells were counted within a 100 μm × 100 μm area in the SN. Only neurons with clearly visible nucleoli were counted.

### Measurement of locomotor activity

Total locomotor activity (ambulation and rearing) was measured using a computerized Actophotometer (IMCORP, India). An array of 16 infrared emitter/detector pairsmeasured animal activity along a single axis of motion, the digital data being displayed on the front panel meter as ambulatory movements. Rats were allowed to acclimatize to the observation chamber for a period of 2 min. The activity was monitored continuously for a period of 5 min. Total locomotor activity is expressed as mean of sum of total ambulatory photo beam counts and total rearing photo beam counts per 5 min per animal [58].

### Motor function test

The effect of dichlorvos on muscle performance was evaluated using rota-rod apparatus. The speed was set at 10 rpm and cut off time was 180 s. All rats were given two initial training trials of 180 s, approximately 10 min apart, to maintain posture on the rota-rod (3 cm in diameter and rotating at a constant 10 revolutions/min). After the initial training trials, a baseline trial of 120 s was conducted. The time each animal remained on the rota- rod was recorded; animals not falling off the rota-rod were given a maximum score of 180 s [59]. Six weeks and 12 weeks after dichlorvos exposure, rats were again tested for endurance performance (three times).

### Open field test

Observations were recorded for three test sessions of 5 min each. Mean of test sessions totals of vehicle and treatment groups ware compared for locomotion (seconds), distance traveled (cm), stereotype events (number) and rearing (number). Open field test was recorded automatically by Video Path Analyzer and soft ware (Anymaze soft ware USA) comprising an open field chamber (50 · 50 · 35 cm), a video camera fixed over the chamber. Test was started 10 s after placing the rat over a black surface at the center of open field chamber.

### Measurement of catalepsy by block method

This scoring method followed is in three steps. Step 1: The rat was taken out of the home cage and placed on a table. If the rat failed to move when touched or pushed gently on the back a score of 0.5 was assigned. Step II: The front paws of the rats were placed alternately on a 3-cm high block. If the rat failed to correct the posture within 15 seconds, a score of 0.5 for each paw was added to the score of step 1. Step III: The front paws of the rat were placed alternately on a 9-cm high block, if the rat failed to correct the posture within 15 seconds a score of 1 for each paw was added to the scores of steps I and II. Thus, the highest score for any animal was 3.5 (cut off score) and that reflects total catalepsy.

### Behavioral assessment by Bar test

Behavioral assessment in dichlorvos induced calaleptic rat was studied by the method of Kulkarni [60]. Cataleptic was measured with a high bar test method. In the bar test, front paws of the rat were gently placed on a horizontal metal bar with 5-6 mm diameter and placed 10 cm above ground level and the length of time, the rats maintained in this abnormal posture with at least one paw was measured. The test was terminated when the paw of animal touched the ground or 180 sec had passed. The total time till which animals stayed on the bar was recorded. Finally, scores at different time points (first day, 6^th ^week, 12^th ^weeks after dichlorvos treatment) were added and expressed as a cumulative catalepsy score for comparison purposes.

### Immunological detection of TH, α- synuclein and synaptophysin

The samples containing 75 μg protein from SN, Hippocampus and CS regions were boiled in laemmli buffer for 5 min and subjected to electrophoresis (12.5% SDS-PAGE for alpha synuclein, and 15% SDS-PAGE for TH and synaptophysin) followed by transfer to a PVDF membrane. The blots were further blocked with 5% non-fat dry milk, the membranes were further incubated with primary anti alpha synuclein/TH antibody/synaptophysin (1:100 Santa Cruz Biotechnology, C.A, USA) at room temperature for 2 hrs. After incubation, the PVDF membranes were washed with PBS plus 0.1% Tween-20 for 30 min, followed by incubation for 1 hr at 37°C with horseradish peroxidase (HRP) conjugated rabbit anti rabbit antibody. After 1 hr of incubation, the blots were again washed with PBS plus 0.1% Tween-20 for 30 min at 5 min interval of time. Immunoreactive proteins were visualized by diaminobenzidine (DAB) from Bangalore Genei. The densitometry analysis of the protein bands ware carried out using SCION IMAGE software (Scion Image Corporation, Fredrick, MD, USA) to compare the relative expression of proteins from different brain regions of dichlorvos treated as well as control rats.

### Immunofluorescence staining of α- synuclein

After sacrificing the rat, brain was isolated, dissected into different sections and washed with normal saline. 5-6 μm thick frozen sections of brain regions were cut by microtome. These were then rinsed three times with phosphate buffer saline (PBS) at 5 min interval. Primary antibody (1:50) was added and the slides were incubated for 1 hr at 37°C. Following incubation, the slides were washed in PBS three times for 5 min each, then the secondary antibody (FITC labeled) diluted in the ratio of 1:30 was added and the slides were incubated for 30 min at 37°C. Again these were washed three times for 5 min each in PBS. The slides were then mounted with glycerol and kept in the dark. Fluorescence was visualized under a fluorescence microscope and the images were recorded.

### RNA extraction and RT-PCR of TH, DBH and α-synuclein

Total RNA was extracted from different regions of rat brain by using the RNA extraction kit (Taurus Scientific, USA) according to manufacturer's instructions. RNA was reverse transcribed in a total volume of 20 μl (RevertAid cDNA synthesis kit, Fermentas) according to manufacturer's instructions. cDNA products (1 μl) were subjected to semi quantitative PCR analysis on a gradient thermal cycler instrument. TH forward (5'-TTCCCCATGTTCAACGGACC-3') and TH reverse (5' GCGAGCACAGTAATCACCTTC-3'). α-synuclein forward (5'TGCTGTGGATATTGTTGTGG3') α-synuclein reverse (5'AGGTGCGGTAGTCTCATGCTC3') DBH forward (5' TTCCCCATGTTCAACGGACC3') and DBH reverse (5'GCGAGCACAGTAATCACCTTC3') primers ware used for amplification. GAPDH, which was used as internal control, was also amplified with GAPDH forward (5' CACTGTGCCCATCTATGAGGG3') and GAPDH reverse (5'TCCACATCTGCTGGAAGGTGG3') primers. PCR cycle of TH comprised of initial denaturation at 94°C for 2 min. The amplification was then carried out for 35 cycles consisting 45 sec each for 94°C (denaturation), 57.5°C (annealing) and 72°C, 1 min (elongation). Final extension was done at 72°C for 10 min. PCR cycle of DBH comprised of initial denaturation at 94°C for 2 min. The amplification was then carried out for 35 cycles consisting 45 sec each for 94°C (denaturation), 54.4°C (annealing) and 72°C, 1 min (elongation). Final extension was done at 72°C for 10 min. For amplification of α synuclein PCR cycle comprised of initial denaturation at 94°C for 1 min. The amplification was then carried out for 35 cycles at 94°C, 45 sec (denaturation), 58°C, 47.9 s (annealing) and 72°C, 1 min (elongation). Final extension was done at 72°C for 10 min. GAPDH, which was used as internal control, was amplified to determine the densitometric analysis of the products using SCION IMAGE software (Scion Image Corporation, Fredrick, MD, USA) to compare the relative mRNA expression of various genes from different brain regions of dichlorvos treated and control rats.

### Protein determination

Mitochondrial protein was determined by the method of Lowry et al [61] using bovine serum albumin as standard.

### Statistical analysis

Data were expressed as mean ± S.D. and statistically significant differences between control and treatment were determined by Student's t-test.

## Abbreviations

(PD): Parkinson's disease; (SNpc): Substantia nigra pars compacta; (DDVP): dichlorvos; (OP): organophosphate; (ETC): Electron transfer chain; (MPTP): N-methyl-4-phenyl-1,2,3,6-tetrahydropyridine; (SN): Substantia nigra; (CS): Corpus striatum; (ROS): Reactive oxygen species; (MPP):1-methyl-4-phenyl-2,3- dihydropyridinium; (MDA): Malondialdehyde; (TH): Tyrosine Hydroxylase; (Mn-SOD): Mn Superoxide Dismutase; (DBH): Dopamine -β - hydroxylase; (DA): dopamine; (HVA): Homovanillic acid.

## Competing interests

The authors declare that they have no competing interests.

## Authors' contributions

Author contributions: K.D and BK., designed research. BK. performed research; A.B. analyzed EM data; BK and K.D. prepared the manuscript.R.K have been involved in drafting the manuscript. All authors read and approved the final manuscript

## Supplementary Material

Additional file 1 **Effect of chronic dichlorvos exposure on Mn SOD activity in substantia nigra and corpus striatum of rat brain**. Dichlorvos treated rats received 2.5 mg/kg b.wt of dichlorvos, sc., for 12 weeks and control animals received equal volume of corn oil. The values are mean ± SD of 6 animals in each group. **P < .0.01, significantly different from controls SN.*P < .0.05, significantly different from controls CS. SN: substantia nigra; CS: corpus striatum. Click here for file

Additional file 2 **Effect of chronic dichlorvos exposure on Dopamine beta hydroxylase activity in substantia nigra and corpus striatum of rat brain**. Dichlorvos treated rats received 2.5 mg/kg b.wt of dichlorvos, sc., for 12 weeks and control animals received equal volume of corn oil. The values are mean ± SD of 6 animals in each group. NS-Non significant. SN: substantia nigra; CS: corpus striatum. Click here for file

Additional file 3 **Effect of chronic dichlorvos exposure on Monoamine oxidase B activity in substantia nigra and corpus striatum of rat brain**. Dichlorvos treated rats received 2.5 mg/kg b.wt of dichlorvos, sc., for 12 weeks and control animals received equal volume of corn oil. The values are mean ± SD of 6 animals in each group. NS-Non significant.SN: substantia nigra; CS: corpus striatum. Click here for file

Additional file 4 **Effect of chronic dichlorvos exposure on Acetylcholinesterase activity in substantia nigra and corpus striatum of rat brain**. Dichlorvos treated rats received 2.5 mg/kg b.wt. of dichlorvos; sc, for 12 weeks and control animals received equal volume of corn oil. Ns-nonsignificant. Click here for file

Additional file 5 **a&b. Effect of dichlorvos on cataleptic behavior (Bar test and Block test)**. Dichlorvos treated rats received 2.5 mg/kg b.wt. of dichlorvos; sc, for 12 weeks and control animals received equal volume of corn oil. *p < 0.05 significantly different from control group. Click here for file
