# Supplementary material for: Nigrostriatal neuronal death following chronic dichlorvos exposure: crosstalk between mitochondrial impairments, α synuclein aggregation, oxidative damage and behavioral changes
Source: Mol Brain. 2010 Nov 13;3:35. doi: 10.1186/1756-6606-3-35 (PMC2996378; doi:10.1186/1756-6606-3-35)
Supplement: Additional file 1 — Effect of chronic dichlorvos exposure on Mn SOD activity in substantia nigra and corpus striatum of rat brain. Dichlorvos treated rats received 2.5 mg/kg b.wt of dichlorvos, sc., for 12 weeks and control animals received equal volume of corn oil. The values are mean ± SD of 6 animals in each group. **P < .0.01, significantly different from controls SN.*P < .0.05, significantly different from controls CS. SN: substantia nigra; CS: corpus striatum. [file 1756-6606-3-35-S1.DOCX]

#####

|  | **MnSOD activity (U/mg protein)** | |
| --- | --- | --- |
|  | **Control group** | **Dichlorvos Treated**  **(2.5 mg/kg b. wt)** |
| **SN**  **CS** | 25.78 + 5.27  30.08 +5.2 | 12.5+ 3.5^**^  19.94+ 3.67^*^ |

##### Additional file 1. Effect of chronic dichlorvos exposure on Mn SOD activity in substantia nigra and corpus striatum of rat brain
