# Supplementary material for: Nigrostriatal neuronal death following chronic dichlorvos exposure: crosstalk between mitochondrial impairments, α synuclein aggregation, oxidative damage and behavioral changes
Source: Mol Brain. 2010 Nov 13;3:35. doi: 10.1186/1756-6606-3-35 (PMC2996378; doi:10.1186/1756-6606-3-35)
Supplement: Additional file 2 — Effect of chronic dichlorvos exposure on Dopamine beta hydroxylase activity in substantia nigra and corpus striatum of rat brain. Dichlorvos treated rats received 2.5 mg/kg b.wt of dichlorvos, sc., for 12 weeks and control animals received equal volume of corn oil. The values are mean ± SD of 6 animals in each group. NS-Non significant. SN: substantia nigra; CS: corpus striatum. [file 1756-6606-3-35-S2.DOCX]

|  | **Dopamine beta hydroxylase activity**  **(U/mg protein)** | |
| --- | --- | --- |
|  | **Control group** | **Dichlorvos Treated**  **(2.5 mg/kg b. wt)** |
| **SN**  **CS** | 10.20±4.71  15.088±6.56 | 9.88±4.22**^NS^**  14.54+ 5.29 ^NS^ |

Additional file 2. **Effect of chronic dichlorvos exposure on Dopamine beta hydroxylase activity in substantia nigra and corpus striatum of rat brain**
