# Supplementary material for: Nigrostriatal neuronal death following chronic dichlorvos exposure: crosstalk between mitochondrial impairments, α synuclein aggregation, oxidative damage and behavioral changes
Source: Mol Brain. 2010 Nov 13;3:35. doi: 10.1186/1756-6606-3-35 (PMC2996378; doi:10.1186/1756-6606-3-35)
Supplement: Additional file 3 — Effect of chronic dichlorvos exposure on Monoamine oxidase B activity in substantia nigra and corpus striatum of rat brain. Dichlorvos treated rats received 2.5 mg/kg b.wt of dichlorvos, sc., for 12 weeks and control animals received equal volume of corn oil. The values are mean ± SD of 6 animals in each group. NS-Non significant.SN: substantia nigra; CS: corpus striatum. [file 1756-6606-3-35-S3.DOCX]

|  | **Monoamine oxidase B activity** **(U/mg protein)** | |
| --- | --- | --- |
|  | **Control group** | **Dichlorvos Treated**  **(2.5 mg/kg b. wt)** |
| **SN**  **CS** | 6.57±0.33  9.91±0.12 | 7.79±0.23 **^NS^**  10.42+ 0.28 **^NS^** |

**Additional file 3**. **Effect of chronic dichlorvos exposure on Monoamine oxidase B activity in substantia nigra and corpus striatum of rat brain**
