# Supplementary material for: Nigrostriatal neuronal death following chronic dichlorvos exposure: crosstalk between mitochondrial impairments, α synuclein aggregation, oxidative damage and behavioral changes
Source: Mol Brain. 2010 Nov 13;3:35. doi: 10.1186/1756-6606-3-35 (PMC2996378; doi:10.1186/1756-6606-3-35)
Supplement: Additional file 4 — Effect of chronic dichlorvos exposure on Acetylcholinesterase activity in substantia nigra and corpus striatum of rat brain. Dichlorvos treated rats received 2.5 mg/kg b.wt. of dichlorvos; sc, for 12 weeks and control animals received equal volume of corn oil. Ns-nonsignificant. [file 1756-6606-3-35-S4.DOCX]

|  | **AChE (nmol product formed / min/ mg protein)** | |
| --- | --- | --- |
|  | **Control group** | **Dichlorvos Treated**  **(2.5 mg/kg b. wt)** |
| **SN**  **CS** | 135 ± 15.60  129±14.32 | 132 ± 8.20 ^NS^  124+16.28 ^NS^ |

Additional file 4. Effect of chronic dichlorvos exposure on Acetylcholinesterase activity in substantia nigra and corpus striatum of rat brain
