# Supplementary material for: Nigrostriatal neuronal death following chronic dichlorvos exposure: crosstalk between mitochondrial impairments, α synuclein aggregation, oxidative damage and behavioral changes
Source: Mol Brain. 2010 Nov 13;3:35. doi: 10.1186/1756-6606-3-35 (PMC2996378; doi:10.1186/1756-6606-3-35)
Supplement: Additional file 5 — a&b. Effect of dichlorvos on cataleptic behavior (Bar test and Block test). Dichlorvos treated rats received 2.5 mg/kg b.wt. of dichlorvos; sc, for 12 weeks and control animals received equal volume of corn oil. *p < 0.05 significantly different from control group. [file 1756-6606-3-35-S5.DOCX]

|  | **Control (s)** | **Dichlorvos(s)** |
| --- | --- | --- |
| First day | 6.167±0.7792 | 7.343±0.753 |
| 6^th^ week | 5.134±0.986 | 147.66±0.138* |
| 12^th^ week | 5.567±0.531 | 151.32±0.862* |

**Additional file 5a Effect of dichlorvos on cataleptic behavior (Bar test)**

|  | **Control (score)** | **Dichlorvos (score)** |
| --- | --- | --- |
| **First day** | 0.00±0.0 | 0.500±0.753 |
| **6^th^ week** | 0.00±0.0 | 1.40±0.138* |
| **12^th^ week** | 0.00±0.0 | 2.032±0.862* |

**Additional file 5b. Effect of dichlorvos on cataleptic behavior (Block test)**
